# Role of nitric oxide in *Salmonella typhimurium*-mediated cancer cell killing

**DOI:** 10.1186/1471-2407-10-146

**Published:** 2010-04-17

**Authors:** Yoram Barak, Frank Schreiber, Steve H Thorne, Christopher H Contag, Dirk deBeer, A Matin

**Affiliations:** 1 Department of Microbiology and Immunology, Sherman Fairchild Science Building, Stanford University School of Medicine, 299 Campus Drive, Stanford, CA 94305, USA; 2 Microsensor Research Group, Max Planck Institute for Marine Microbiology, Celsiusstr. 1 D-28359 Bremen, Germany; 3 Department of Pediatrics and Bio-X Program, James H. Clark Center, Stanford University School of Medicine, 318 Campus Drive, Stanford, CA 94305, USA; 4 Department of Surgical Oncology, Hillman Cancer Center, Centre Avenue, University of Pittsburgh, Pittsburgh, PA 15232, USA; 5 Codexis Inc. 200 Penobscot, Redwood City, CA 94063, USA

## Abstract

**Background:**

Bacterial targeting of tumours is an important anti-cancer strategy. We previously showed that strain SL7838 of *Salmonella typhimurium *targets and kills cancer cells. Whether NO generation by the bacteria has a role in SL7838 lethality to cancer cells is explored. This bacterium has the mechanism for generating NO, but also for decomposing it.

**Methods:**

Mechanism underlying *Salmonella typhimurium *tumour therapy was investigated through *in vitro *and *in vivo *studies. NO measurements were conducted either by chemical assays (*in vitro*) or using Biosensors (*in vivo*). Cancer cells cytotoxic assay were done by using MTS. Bacterial cell survival and tumour burden were determined using molecular imaging techniques.

**Results:**

SL7838 generated nitric oxide (NO) in anaerobic cell suspensions, inside infected cancer cells *in vitro *and in implanted 4T1 tumours in live mice, the last, as measured using microsensors. Thus, under these conditions, the NO generating pathway is more active than the decomposition pathway. The latter was eliminated, in strain SL7842, by the deletion of *hmp*- and *norV *genes, making SL7842 more proficient at generating NO than SL7838. SL7842 killed cancer cells more effectively than SL7838 *in vitro*, and this was dependent on nitrate availability. This strain was also ca. 100% more effective in treating implanted 4T1 mouse tumours than SL7838.

**Conclusions:**

NO generation capability is important in the killing of cancer cells by *Salmonella *strains.

## Background

Strains of *Salmonella typhimurium *and other bacteria tend to target tumours and have been used for cancer therapy and as vehicles of gene delivery in enzyme prodrug therapy (GDEPT) [1-3]. In our previous GDEPT work with a number of reductive prodrugs, including our newly discovered *6-chloro-9-nitro-5-oxo-5H-benzo [a]phenoxazine *(CNOB), we employed *S. typhimurium *strain SL7838 (Δ*sopE *and Δ*aroA*) to deliver the *chrR6 *gene to tumours. SL7838 is attenuated with enhanced preference for tumour localization, and the *chrR6*-encoded enzyme (ChrR6) possesses superior capacity to activate reductive prodrugs [4,5]. The treatments generated a dual killing effect of tumour cells, one due to the SL7838 bacteria themselves, and the other to the activated prodrugs [4,5].

Here we investigated the mechanism of cancer cell killing by SL7838. It is thought that competition for nutrients by bacteria plays a role. However, bacteria may also be able to produce substances that are toxic to cancer cells. One such candidate is the nitric oxide free radical molecule (NO). That this radical has anticancer activity is shown by the fact that high expression levels of mammalian nitric oxide synthase (iNOS), which generates NO, or the external provision of this molecule (e.g. by nitro-aspirin) arrest tumour proliferation [6-9]. Like other *S. typhimurium *strains, SL7838 possesses nitrate and nitrite reductases and is thus able to convert nitrate and nitrite to NO under oxygen limitation [10]. As nitrate and nitrite are present in mammalian body (30-150 and 0.3-20.0 μM, respectively [11]), and tumours contain hypoxic regions [4,12-15], SL7838 has the potential to generate NO in tumours. However, this bacterium also possesses the Hmp and NorV enzymes that decompose NO in order to detoxify it. Thus, its ability to generate NO would depend on the relative activity of these opposing pathways under given conditions. We examined the capacity of SL7838 to generate NO in a variety of settings and report that NO generation is likely to play a major role in its lethality to cancer cells.

## Methods

### Bacterial strains, plasmids primers and chemicals

See **Table S1**. *Salmonella typhimurium *strain SL7838 was transformed by electroporation [16] with plasmids pCGSL1, encoding the Lux operon, and pFVP25.1, encoding ChrR6. Deletion of the *norV, hmP *genes (to generate strain SL7842) was carried out as described [17], using the specified primers and plasmids (Additional file 1); deletions were confirmed by three independent PCR assays. The NO donor, *2-(N,N-Diethylamino)-diazenolate 2-oxide *(DEA NONOate); nitrate, nitrite hemoglobin and sodium dithionite were purchased from Sigma Inc. MO; Griess reagent (for NO measurement), *4-amino-5-methylamino-2',7' difluorofluorescein diacetate *(DAF-FM di-acetate) from Molecular Probe Inc. CA; and SensoLyte™ caspase assay kit were from AnaSpec Inc. CA.

### Tumour cell lines and viability assays

JC (murine mammary cancer) cells were obtained from Cancer Research UK; 4T1 (murine mammary cancer) and A-2780 (human ovarian cancer) and 293T (human kidney cancer) from ATCC. MDA-MDB-435 (human breast cancer) was kindly supplied by Dr. Gambhir. All cells were grown as adherent cultures in DMEM medium supplemented with 10% FBS and 1% pen/strep. Cell viability was measured by MTS assay, as described [4].

### Cancer cell infection and chemical exposure

Confluent cancer cells were infected with the appropriate bacterial strain [MOI, 10; 4], and after incubation (1 h, 37°C) were washed twice with preheated DMEM and further incubated in DMEM plus gentamycin (1 h, 37°C; 100 μg/ml). Following another wash, the cells were suspended in DMEM-gentamycin (10 μg/ml) and exposed to NO_3_^- ^(150 μM), NO_2_^- ^(2 μM), or the NO donor DEA NONOate (final NO concentration, 0.2 μM).

### Probing intracellular NO

Washed cell were suspended in PBS buffer (50 μl) supplemented with DAF-FM-diacetate (5 μM) and incubated in the dark for 15 min. 5 μl were spotted on a microscope slide, air dried in the dark, and after the addition of 5 μl Vectashield^® ^fluorescence anti-quenching medium, were covered by a glass cover slip. DAF-FM reacts quantitatively with NO to form a fluorescent benzotriazole derivative. Cells were visualized at 1000× magnification (Olympus B×60 upright fluorescence microscope), imaged (Hamamatsu Orca1 CCD), and pseudocolored, with identical settings for each image (Image Pro Plus 5.0).

### Oxygen and nitric oxide quantification in implanted tumours in live mice

O_2 _and NO profiles of tumours were determined in groups of two mice, one with uninfected, the other infected with SL7838; measurements were made at three individual regions of each tumour (which had an average depth of 14 mm). Clark-type NO and O_2 _microsensors were used [18,19]. Microsensors were connected to a picoammeter and polarized at +750 and -800 mV for NO and O_2 _measurements, respectively. Amperometric responses were recorded with DAQCard-AI-16XE-50 (National Instruments, Austin, TX) data acquisition system, using the μ-profiler (Lubos Ploerecky, MPI Bremen, Germany) software at 1s intervals. Each recording represented an average of 2,000 signals at 10 kHz frequency. The microsensors were mounted on a 3-axis motorized micromanipulator (MM 33; Märzhäuser, Wetzlar, Germany) controlled by μ-profiler software (3564-K-024-BC; Faulhaber Group, Schönaich, Germany) for μ-positioning. Tumours were overlaid with low melt agarose (4%, Sigma, Inc.), using a transparent plastic ring. The microsensor tip was placed at the surface of the tumour manually using a stereo microscope (Omano, Inc). The profiles were measured beginning from the inside of the tumour. The NO sensor has a sensitivity range of ~2 - 5 pA μM^-1 ^and a spatial resolution of 60-80 μm; it was calibrated as described [18].

### Treatment of implanted tumours

4T1 tumours were implanted in immunocompetent BALB/c mice as before [5] - s.c.; 10^6 ^cell inoculum. The resulting tumours (10-14 day post inoculation) were injected with PBS, SL7838, or SL7842 (10^5^CFU). Tumour burden (by caliper) and the animal weight were determined at appropriate intervals. The tumour and the bacterial cells were visualized as before [5] via luminescence due to firefly luciferase and Lux expression, respectively. Imaging of bacterial Lux activity in anesthetized (2% isoflurane) mice was done in an IVIS Spectrum system (Caliper, CA). In addition, imaging was performed 10 min after intraperitoneal injection of luciferin (150 μL of 30 mg/ml solution). The signal generated after luciferin addition (Luc expression) includes the signal due to the bacterial Lux expression, but since the former was more than 50 fold greater, the latter is negligible. All animal studies were performed according to approved Stanford's IACUC and biosafety committee protocols.

### Statistical Analyses

Student T tests were performed in all statistical analysis, except for the Kaplan-Meier survival curves, where Logrank tests were used. P-values are indicated; values of less than 0.05 were considered significant.

## Results

**Figure 1:**
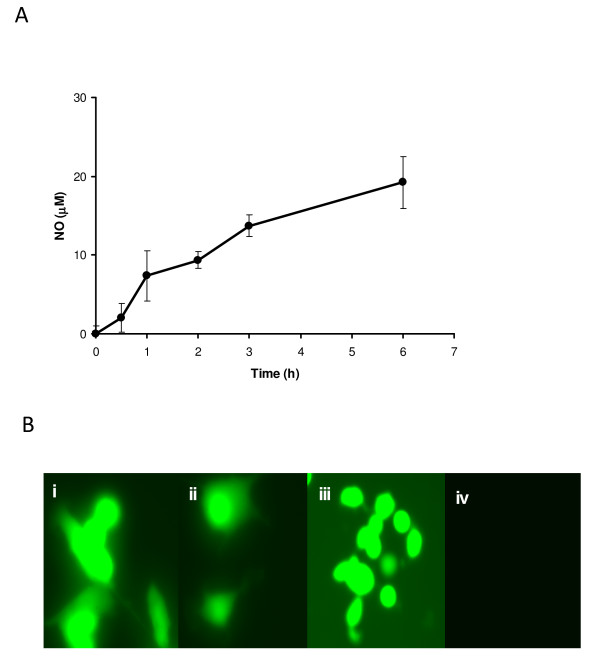
**A. NO production by SL7838 cell suspensions (A_660_, 1.5; glucose-M9 medium) incubated without shaking in the presence of nitrate**. NO concentration was determined by the oxy-haemoglobin method [24]; **B**. NO (green fluorescence) production in SL7838-infected JC cells as measured by DAF-FM diacetate. The mammalian NO synthase inhibitor, L-NAME (20 μM), was added to JC cell growth medium 24 h prior to bacterial infection. SL7838-infected JC cells were supplemented with 0.2 μM NO donor (DEA NONOate) positive control (i), 150 μM NO_3_^- ^(ii), or 2 μM NO_2_^- ^(iii), or none of these compounds (iv). Uninfected JC cells receiving 150 μM NO_3_^- ^also did not generate the fluorescence, as in (iv). The experiment was run in triplicate. Error bars represent STDEV.

We determined whether SL7838 cell suspensions can generate NO. When supplied nitrate (500 μM) under partial anaerobiosis, the suspensions generated NO for several hours (Figure 1A). We next determined if SL7838 can generate NO after infecting JC murine cancer cells. L-NAME (20 μM) was used to inhibit the indigenous NO generating activity of JC cells (due to iNOS), and NO formation was detected by DAF-FM diacetate; the latter fluoresces in the presence of NO, as we confirmed experimentally (Figure. 1BiA). Addition of nitrate or nitrite (at serum concentrations) to SL7838-infected JC cells generated the fluorescence signal (Figure. 1Bii and 1Biii). Similar results were obtained with infected human MDA-MB-435 breast, or A2780 ovarian cancer cells (not shown).

**Figure 2:**
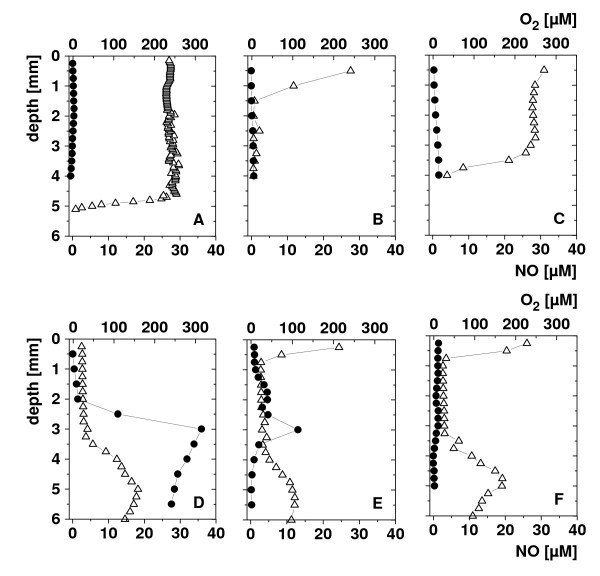
***In vivo *measurement of NO, and O_2 _in dorsal 14-day old 4T1 subcutaneous tumours in BALB/c mice (50-100 mm^3-^)**. Symbols: Panels A-C: vertical NO (black circle) and oxygen (white triangle) concentration profiles in 3 different spots of an uninfected tumour; Panels D-F: such profiles in a tumour that was infected intratumourally with the bacteria 24 h prior to the measurement. Amperometric microsensors were used; representative results are presented.

We then explored NO generation directly in implanted s.c. 4T1 tumours in living mice after intratumoural administration of SL7838. The tumours were impaled with a microelectrode for NO measurement [18,19]; an oxygen microelectrode measured oxygen concentration in parallel. The tumours were examined at three separate locations. In the uninfected control tumours, no NO generation was seen, and the oxygenation levels changed with tumour depth as well as location (Figure. 2, **panels A-C**) revealing, as expected, several regions of anoxia (<~1 μM oxygen). In the infected tumours (Figure. 2, **panels D-F**), NO generation of up to 300 μM was seen (region D); the amount varied in different regions (e.g., lower levels in region E; none in F). Anaerobiosis tended to be more marked in the infected tumours, but there was no obvious correlation between the tumour oxygenation status and NO production.

**Figure 3:**
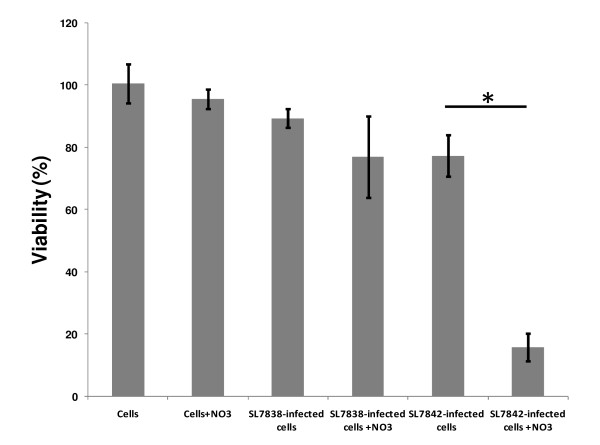
**Loss of viability at 48 h of 4T1 breast cancer cells *in vitro***. Viability loss at 48 h of 4T1 breast cancer cells *in vitro *with no additions ("cells") and as a result of NO_3_^- ^(150 μM) addition with or without infection by strain SL7838, or SL7842, as indicated (n = 3; error bars indicate standard deviation, *p <*0.0002*).

In order to test if NO generation by SL7838 contributes to its lethality to cancer cells, we generated strain SL7842, which was rendered more active in NO generation by deleting the *norV *and *hmp *genes that encode the pathways for decomposing this radical; while SL7838 cell suspensions caused complete disappearance of added NO (90 μM) within ca. 200 min (as measured by Greiss assay), those of SL7842 generated no decomposition; the NO donor, DEA NONOate, was used in these experiments. SL7848-infected 4T1 cells exhibited much greater loss of viability than those infected with SL7838 (Figure. 3; compare bars 4 and 6 from the left). The killing of the cancer cells was dependent both on nitrate availability and bacterial infection. Invasion/replication assays showed that both the strains were equally competent in invading and replicating in cancer cells (not shown). Similar results were obtained with JC, A2780, or 293T cancer cells (not shown).

**Figure 4:**
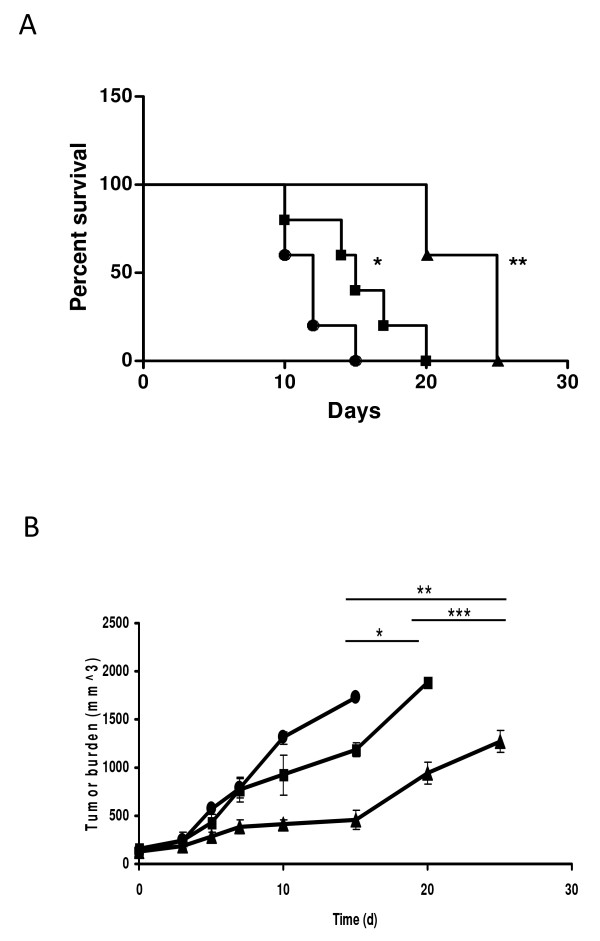
**Efficacy of bacterial treatment of implanted cancer in mice**. **A**. Survival (Kaplan-Meier) curves of BALB/c mice bearing s.c. 4T1 tumours (50-100 mm^3^). Symbols: untreated mice administered saline (control; black circle); SL7838-treated mice (black square); SL7842-treated mice (black triangle) (n = 5 animals/group) (*p <0.015, **p < 0.003). Bacteria (10^5 ^CFU) were administered intratumourally at the start of the experiment. **B**. Tumour burden measured at indicated time points using caliper. Symbols as above. T-tests *p < 0.064, **p < 0.022, ***p < 0.035

Strain SL7842 proved more effective also in treating implanted 4T1 tumours in mice. Following the development of subcutaneous (s.c.) tumours (10 - 14 days post inoculation of 10^6 ^4T1 cells [5]), separate groups of mice were injected intratumourally with saline (control), strain SL7838, or SL7842. While treatment with SL7838 did increase survival (Figure. 4A; Log Rank Test, *p <*0.01*) as reported before, treatment with SL7842 generated ~100% greater survival (Log Rank Test, **p <*0.003*). Similar outcome is evident from tumour burden measurements (Figure. 4B). The bacteria remained localized to the tumour (not shown), as revealed by bioluminescence imaging as we previously reported ([5]; see Methods); and the mice exhibited no significant change in weight, suggesting non-toxicity of the treatment.

## Discussion

*Salmonella *bacteria are known to kill infected cancer cells [5,21,22]. These bacteria have the capacity to generate NO, but also possess a pathway to decompose it. Since NO is a known anti carcinogen [6-9], this investigation addressed two questions, whether *Salmonella *strain SL7838 can generate NO under conditions found inside the cells and within the tumours; and whether this potential capacity has a role in cancer treatment by these bacteria. Cell suspensions of this bacterium under partial anaerobiosis generated NO, indicating that the pathway for the formation of this radical is more active than that involved in its decomposition. A similar result was seen within several cancer cell lines infected with this bacterium. NO was generated in the presence of LNAME, ensuring that the mammalian iNOS was rendered inactive, and no NO was generated by nitrate-supplemented uninfected cancer cells, or the infected cancer cells not exposed to nitrate (Figure. 1Biv). Thus, the intracellular NO seen in these experiments was of bacterial origin and contingent on nitrate availability. This result also shows that *Salmonella *infecting these cells experience anaerobiosis, since oxygen is their preferred electron acceptor, and they generate NO from nitrate (or nitrite) only under anaerobic conditions.

We further investigated whether NO generation occurred within the implanted 4T1 tumours in mice using micro sensors for both NO and oxygen. The electrodes were impaled in the tumours and measurements were made in several locations. NO generation can occur within the tumours regardless of bacterial infection because of mammalian nitric oxide synthases. Of these three such enzymes, nNOS and eNOS are constitutive but generate very low levels of NO. The enzyme responsible for most NO generation is iNOS, which requires induction by cytokines [20]. However, no NO was detected in the uninfected tumours, indicating lack of activity of these enzymes. Substantial NO was seen within the tumours infected by the bacteria, showing once again that the bacterial infection was the cause of NO generation. Both the distribution of NO as well as of oxygen within the tumours was highly uneven as might be expected form previous findings of tumour heterogeneity and the fact that intratumourally injected SL7838 remain confined to localized tumour regions [5].

The above results affirmed the first query of this investigation, i.e., that *Salmonella *SL7838 does indeed generate NO under conditions found within cancer cells and tumours. The second question concerning the role of NO in lethality was addressed by generating strain SL7848 with deactivated NO decomposing pathway. This strain was more active in killing cancer cells *in vitro*. Furthermore, it had a vastly greater capacity to treat implanted 4T1 tumours in mice compared to strain SL7838 which because it retains the NO decomposition pathway has lower capacity to generate NO. We conclude that NO generation plays an important role in anticancer activity of *Salmonella *bacteria, and that enhancement of this capacity can benefit cancer therapy utilizing these agents by themselves or as vehicles of gene delivery.

## Conclusions

*Salmonella *stains with enhanced capacity for NO generation are likely to prove beneficial in anti-cancer strategies aimed at using these bacteria for gene delivery and cancer treatment. Since many other bacteria can also reduce NO_3_^- ^and NO_2_^- ^[23], this approach may prove to be generally beneficial in anti-cancer measures involving bacteria.

## Competing interests

The authors declare that they have no competing interests.

## Authors' contributions

YB constructed the mutants, designed and conducted many of the experiments, and wrote along with FS first draft of the manuscript. FS & DdB performed NO and oxygen measurements in implanted tumours. SHT & CHC carried out molecular imaging and statistical analysis. AM, along with YB, conceived the study, participated in its design and coordination and put together the final manuscript version. All authors read and approved the final manuscript.

## Pre-publication history

The pre-publication history for this paper can be accessed here:

http://www.biomedcentral.com/1471-2407/10/146/prepub

## Supplementary Material

Additional file 1 **Bacterial strains, plasmids, and primers**. All bacterial strains and molecular tools used in this study. Click here for file
