# Supplementary material for: Role of nitric oxide in Salmonella typhimurium-mediated cancer cell killing
Source: BMC Cancer. 2010 Apr 17;10:146. doi: 10.1186/1471-2407-10-146 (PMC2868810; doi:10.1186/1471-2407-10-146)
Supplement: Additional file 1 — Bacterial strains, plasmids, and primers. All bacterial strains and molecular tools used in this study. [file 1471-2407-10-146-S1.DOCX]

# Additional files

**Additional file 1:** Bacterial strains, plasmids, and primers

|  | **Relevant characteristics** | **Reference or source** |
| --- | --- | --- |
| **strains** |  |  |
| *S. typhimurium* SL7838 | Attenuated strain containing *aroA* and *sopE* gene deletions | [4] |
| SL7842 | SL7838 with deletion of  *norV & hmp* | This research |
| **Plasmids** |  |  |
| pCGLS1 | Containing Luciferase gene allowing in-vivo imaging | [22, 23] |
| pKD3 | Cloning vector encoding chloramphoenicol resistance cassette | [20] |
| pKD46 | Red recombinase expression vector | [20] |
| **Primers** |  |  |
| F*norV* | ggtggttatgtctattctggttaaaaataatattcattgggttggTGTGTAGGCTGGAGCTGCTTC | This research |
| R*norV* | ccgactcattttgcctccgtcgccagtacgtcgaacacgtctttaCATATGAATATCCTCCTT | This research |
| F*hmp* | gagtctgacgaatttcaacgggatttcttttcagctttgtgTGTGTAGGCTGGAGCTGCTTC | This research |
| R*hmp* | TCGCCTTAACGATAACGCCCGTTTTTTCAGAGGATTTGTTGCAACATATGAATATCCTCCTT | This research |
